# One-step affinity purification of fusion proteins with optimal monodispersity and biological activity: application to aggregation-prone HPV E6 proteins

**DOI:** 10.1186/s12934-018-1039-z

**Published:** 2018-12-01

**Authors:** Anna Bonhoure, Auguste Demenge, Camille Kostmann, Leticia San José, Eva De la Cal, Pilar Armisen, Yves Nominé, Gilles Travé

**Affiliations:** 1 Équipe Labellisée Ligue 2015, Department of Integrated Structural Biology, Institut de Génétique et de Biologie Moléculaire et Cellulaire (IGBMC), INSERM U1258/CNRS UMR 7104/Université de Strasbourg, 1 rue Laurent Fries, BP 10142, 67404 Illkirch, France; 2 ABT-Agarose Bead Technologies, C/La Forja, 9, Torrejón de Ardoz, 28850 Madrid, Spain

**Keywords:** Protein aggregation, Protein solubility, MBP fusion, IMAC, One-step purification

## Abstract

**Background:**

Bacterial expression and purification of recombinant proteins under homogeneous active form is often challenging. Fusion to highly soluble carrier proteins such as Maltose Binding Protein (MBP) often improves their folding and solubility, but self-association may still occur. For instance, HPV E6 oncoproteins, when produced as MBP-E6 fusions, are expressed as mixtures of biologically inactive oligomers and active monomers. While a protocol was previously developed to isolate MBP-E6 monomers for structural studies, it allows the purification of only one MBP-E6 construct at the time. Here, we explored a parallelizable strategy more adapted for biophysical assays aiming at comparing different E6 proteins.

**Results:**

In this study, we took advantage of the distinct size and diffusion properties of MBP-E6 monomers and oligomers to separate these two species using a rapid batch preparation protocol on affinity resins. We optimized resin reticulation, contact time and elution method in order to maximize the proportion of monomeric MBP-E6 in the final sample. Analytical size-exclusion chromatography was used to quantify the different protein species after purification. Thus, we developed a rapid, single-step protocol for the parallel purification of highly monomeric MBP-E6 samples. MBP-fused HPV16 E6 samples obtained by this approach were validated by testing the binding to their prototypical peptide targets (the LXXLL motif from ubiquitine ligase E6AP) by BIAcore-SPR assay.

**Conclusions:**

We have designed a rapid single-step batch affinity purification approach to isolate biologically active monomers of MBP-fused E6 proteins. This protocol should be generalizable to isolate the monomer (or the minimal biologically active oligomer) of other proteins prone to self-association.

**Electronic supplementary material:**

The online version of this article (10.1186/s12934-018-1039-z) contains supplementary material, which is available to authorized users.

## Background

Protein solubility is a major issue in recombinant protein purification from *Escherichia coli*. It is influenced by different parameters, such as proper folding and/or aggregation. Under the stress induced by high rates of heterologous protein expression, inactive misfolded polypeptides accumulate as inclusion bodies. They can be solubilized by using chaotropic agents (urea or guanidium hydrochloride) and then refolded in vitro (by dilution or dialysis). However, such an approach can be time-consuming since it requires optimization adapted to every protein, and the yield of final soluble product can be low. Another strategy consists of fusing the protein of interest (also called “passenger protein”) to a solubility-enhancing protein (also called “carrier protein”), such as Thioredoxin, glutathione-S-transferase (GST) or maltose-binding protein (MBP). MBP was reported to be particularly efficient in improving the solubility of its fusion partners [1, 2]. In addition to its ability to stabilize and solubilize the passenger protein, MBP can be used as an affinity tag for purification on amylose resin [3, 4]. However, an MBP-fused protein can sometimes be solubilized in the form of a mixture of properly folded monomers (or minimal biologically active oligomers) and of large oligomers in which the passenger protein is self-associated and its folding and/or biochemical activity may be altered [5–13]. In such situations, the challenge consists in exploring conditions of expression and purification favoring the biologically active monomeric -or minimally oligomeric- MBP-fused samples [9, 11, 14, 15].

E6 proteins produced by oncogenic human papillomaviruses (HPV) are a prototypical case of proteins that display unstable biochemical behavior when produced recombinantly [16–18]. While unfused E6 proteins are expressed in insoluble form, MBP-fused E6 proteins are soluble yet prone to self-association, leading to soluble oligomers, which in turn are unable to specifically interact with protein partners [9, 10, 19]. Furthermore, E6 proteins are generally cysteine-rich. E6 proteins contain two zinc-binding domains, each involving four cysteine residues to coordinate one zinc ion. In addition to these eight cysteine residues highly conserved for structural purposes, E6 proteins contain additional non-conserved cysteine amino acids. For instance, wild-type HPV16 E6 and HPV8 E6 have a total of 14 and 16 cysteine residues, respectively. Oxidation promotes the formation of intermolecular disulfide bridges, and thus increases the aggregative propensity of E6 proteins.

Papillomaviruses E6 oncoproteins establish numerous interactions with host proteins [16–18, 20]. For instance, E6 from “high-risk” mucosal HPV was reported to hijack the ubiquitin ligase E6AP (E6-associated protein), resulting in the proteasome-mediated degradation of the tumor suppressor p53 [21]. Crystal structures of the HPV16 E6 oncoprotein from high-risk mucosal HPV16 in complex with a minimal target fragment from E6AP and the core domain of p53 were solved by X-ray crystallography, using solubility-enhanced mutants of E6 fused to MBP [22, 23].

There are more than 200 HPV types, with different tropisms (mucosal, cutaneous) causing a large variety of phenotypes ranging from warts and condylomas for low-risk HPVs to malignant tumors for high-risk HPVs [24–26]. The present work will focus on E6 oncoproteins from HPV16 and HPV8. HPV16 is the highest-risk mucosal HPV, responsible for more than 50% of cervical cancers and more than 90% of HPV-positive oropharyngeal cancers [27, 28]; whereas HPV8 is a cutaneous HPV type that can generate skin cancers, in particular in individuals with genetic or immunological diseases [29].

Since E6 proteins from different HPV types were reported to have distinct protein interaction preferences by proteomic studies [20], it would be interesting to further decipher their binding properties by quantitative biophysical and structural approaches. The protocol used in our former publications [9–12] allows the isolation of soluble, monomeric E6 proteins by amylose affinity chromatography followed by overnight ultracentrifugation and size-exclusion chromatography (SEC) for the elimination of protein oligomers. Such a strategy is appropriate for structural studies that require relatively large amounts of high quality protein material. However, it allows the purification of only one protein at a time. To decipher the protein–protein interactions of a panel of E6 oncoproteins, parallel purifications would be more appropriate. In addition, a faster purification protocol would reduce the risk of protein aggregation over time.

In the present work, we exploited the distinct size and diffusion properties of monomers and oligomers of MBP-E6 to separate these two species using a rapid batch affinity preparation approach. This led us to obtain a fast, single-step protocol for the preparation of two test E6 oncoproteins under monomeric and biologically active form: HPV16 F47R 4C/4S E6 (a solubility-enhanced mutant of HPV16 E6) and HPV8 E6 (prone to rapid self-association). The long-lasting and non-parallelizable steps of the previous protocol (ultracentrifugation and preparative gel-filtration) were substituted by a fast batch affinity chromatography on reticulated nickel resins. The reticulation state of the resin, the elution protocol and the contact time were adjusted for optimal separation of monomeric and oligomeric MBP-fused E6 proteins. We show that this new and fast protocol is effective enough to obtain soluble and active E6 protein samples suitable for protein–peptide interaction assay as revealed by surface plasmon resonance (SPR). This protocol is amenable to robotization, offering the ability to prepare in a parallel fashion multiple protein samples displaying optimal monodispersity.

## Results

### Protein constructs used for this study

To develop and evaluate a new E6 purification protocol, we used two distinct HPV E6 oncoproteins. On the one hand, HPV16 E6 F47R 4C/4S (thereafter named 16E6mut) is a solubility-optimized mutant of HPV16 E6. The structure of this mutant has been solved by X-ray crystallography and several of its interactions have been precisely characterized by surface plasmon resonance (SPR) and Isothermal Titration Calorimetry [22]. On the other hand, HPV8 E6 (thereafter named 8E6) has a strong tendency to self-associate and is more challenging to purify as a monomer (unpublished observations).

**Fig. 1:**
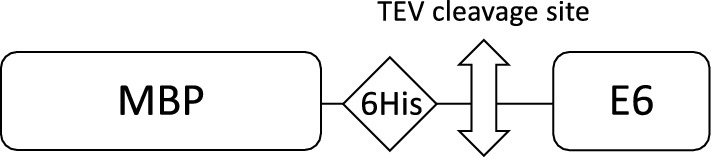
Construct for the production of recombinant HPV E6 protein: MBP is used as a solubilizing fusion while 6His tag allows IMAC on Nickel resin. Purification tags can be removed by TEV protease cleavage

As depicted in Fig. 1, HPV E6 constructs were overexpressed as fusions to the C-terminus of bacterial MBP. MBP is known to favor solubilization of recalcitrant recombinant proteins [1, 2], and can be used for affinity purification on amylose resin. In addition, the constructs include a 6-His tag for immobilized-metal affinity chromatography (IMAC) on Nickel resin and a TEV (Tobacco Etch Virus) protease cleavage site allowing elimination of N-ter purification tags.

### Impact of the reticulation level of affinity resins on the aggregation level of purified MBP-E6 samples

In the protocol published previously for the purification of soluble MBP-E6 protein [9, 10], the initial affinity step was performed on amylose resin. In such conditions, a fraction of the MBP-E6 sample formed oligomers that were detected by static light scattering in a fluorimeter. These soluble oligomers can be eliminated by means of overnight ultracentrifugation followed by preparative gel-filtration [19]. However, this approach requires substantial amounts of protein in order to be efficient and is not suitable for parallel purification of HPV E6 proteins. We hypothesized that testing resins of different reticulation degree might be an alternative strategy to reduce the binding of the oligomeric proteins to the affinity resin since large particles cannot interact with a highly reticulated resin, thus promoting the preferential interaction of small molecules with the affinity resin.

**Fig. 2:**
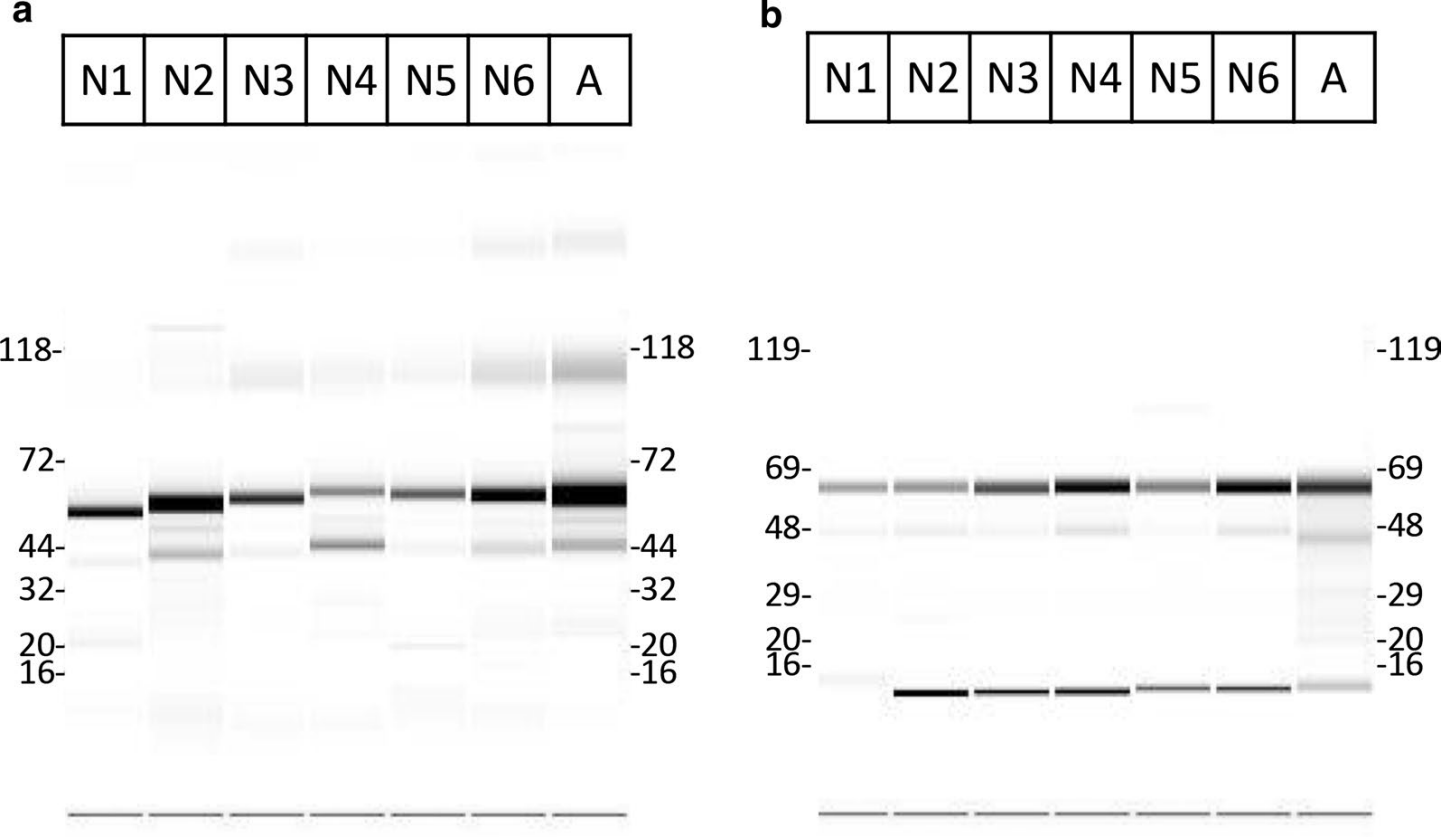
Example of analysis of purified MBP-E6 fusions by microfluidics capillary electrophoresis in denaturing conditions. **a** MBP-16E6mut, **b** MBP-8E6. The data, which normally consist in electropherograms (e.g., curves describing migrating protein signal intensity as a function of molecular size), are plotted as an intensity-based projection to mimic a conventional SDS PAGE display. Molecular weights, obtained from internal standards, are indicated in kDa. A slight shift may sometimes be observed from one lane to another, as observed in (**a**). These eluted samples were obtained after purification on the different resins (N1–6 for the nickel resins; A for amylose resin), 2 h contact time between bacterial lysate and resin, filter elution

Here, we compared the aggregation levels of purified MBP-E6 protein obtained either with the same amylose resin than the one used in the initial protocol (thereafter called A), or with six different reticulated nickel resins (numbered from N1 to N6, as detailed in Table 1). In order to test the preferential binding of the monomer to the different resins, elution was performed by centrifugal filtration on a filter plate to maximize the recovered liquid fraction and the detection of all protein species eluted from the resin. The purity of the final fractions was satisfactory, as checked by microfluidics capillary electrophoresis in denaturing conditions (Fig. 2). The bands corresponding to the protein of interest are visible between the 44 and 72 kDa markers for MBP-16E6mut (expected MW = 61.4 kDa) and between the 48 and 69 kDa markers for MBP-8E6 (expected MW = 60.9 kDa).

**Table 1:** Panel of resins used for affinity purification, gathering technical data provided by the manufacturers

	Manufacturer	Catalogue ref	Reference	Affinity	Nickel ligand	Features	Exclusion limit for globular proteins	Metal ion capacity/binding capacity	Max. pressure
N 1	GE Healthcare	17-5318-06	Ni Sepharose 6 Fast Flow	Nickel	NTA	6% cross-linked agarose	4000 kDa	Approx 15 µmol Ni^2+^/mL gel	0.1 MPa
N 2	ABT Agarose Beads Biotechnologies	10BCL-QHNi-5	High Density Nickel 10 BCL-QHNi-5	Nickel	IDA	10% cross-linked agarose	500 kDa	20–40 µmol Ni^2+^/mL gel	Low pressure
N 3	ABT Agarose Beads Biotechnologies	6BCL-QHNi-2	High Density Nickel 6 BCL-QHNi-2	Nickel	IDA	6% cross-linked agarose	4000 kDa	20–40 µmol Ni^2+^/mL gel	Low pressure
N 4	ABT Agarose Beads Biotechnologies	6BCL-NTANi-2	Nickel NTA agarose resin 6 BCL-NTANi-2	Nickel	NTA	6% cross-linked agarose	4000 kDa	5–19 µmol Ni^2+^/mL gel	Low pressure
N 5	ABT Agarose Beads Biotechnologies	6NiRR-2	Nickel Rapid Run 6 NiRR-2	Nickel	IDA	6% agarose	4000 kDa	5–19 µmol Ni^2+^/mL gel	0.3 MPa
N 6	ABT Agarose Beads Biotechnologies	6NTANiRR-2	Nickel NTA rapid run 6 RR-NTANi-2	Nickel	NTA	6% agarose	4000 kDa	5–19 µmol Ni^2+^/mL gel	0.3 MPa
A	NEB New England Biolabs	E8022S	Amylose resin high flow	Maltose		Cross-linked agarose	Unspecified	7 mg MBP-protein/mL gel	0.5 MPa

**Fig. 3:**
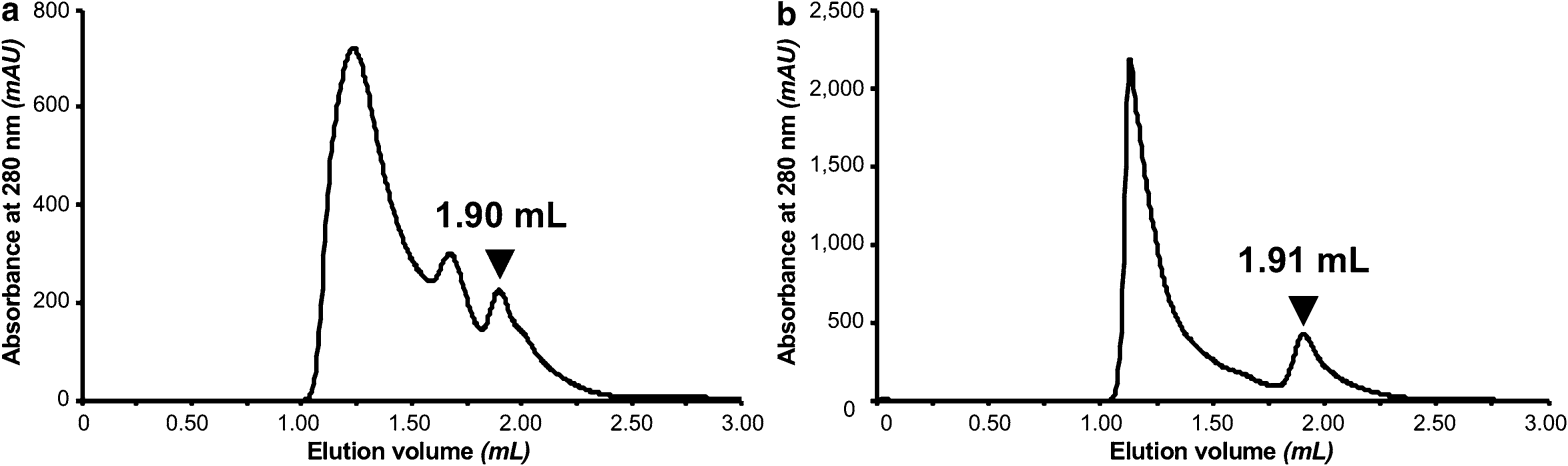
SEC chromatograms of purified MBP-E6 proteins with the initial protocol (amylose resin; 2 h contact time between bacterial lysate and resin; filter elution). The elution peak corresponding to the monomeric protein is indicated by an arrow, according to column calibration using standard proteins. **a** MBP-16E6mut, **b** MBP-8E6

The oligomeric state of the purified protein samples was assessed by analytical SEC, based on absorbance at 280 nm. Figure 3 shows chromatograms of MBP-16E6mut (A) and MBP-8E6 (B) purified using the initial protocol in which the bacterial lysate of overexpressed E6 protein was incubated 2 h with amylose resin. After washing the resin, protein was eluted by maltose-containing buffer and recovered by centrifugal filtration.

According to the column calibration based on (Eqs. 1 and 2), the elution volume for MBP-16E6mut purified on amylose resin (V_e_ = 1.90 mL) corresponds to a molecular weight MW of 75 kDa and a hydrodynamic radius R_h_ of 37 Å. For MBP-8E6, a similar peak appears at an elution volume V_e_ of 1.91 mL, which would correspond to a molecular weight MW = 72 kDa and a hydrodynamic radius R_h_ = 37 Å. Considering the accuracy of the method and the fact that calibration is based on the diffusion properties of globular proteins, we assume that both peaks refer to monomeric MBP-E6 (theoretical molecular weights: MW (MBP-16E6mut) = 61.4 kDa, MW (MBP-8E6) = 60.9 kDa). Since MBP-E6 fusions are not globular [22], the experimental elution volumes are expected to be slightly lower than the calculated elution volume obtained from the calibration (V_e theo_ = 1.95 mL).

For each tested resin, analytical SEC was performed. Based on absorbance at 280 nm, the monomeric and oligomeric protein were detected at a given elution volume. We measured the following elution volumes for monomeric MBP-16E6mut and MBP-8E6 at V_e_ = 1.92 ± 0.01 mL and MBP-8E6: V_e_ = 1.99 ± 0.04 mL, respectively. All peaks with V_e_ < 1.90 mL correspond to particles larger than monomer, that were therefore considered as oligomeric proteins (Fig. 3, Additional file 1). Elution peaks were integrated based on the signal of absorbance at 280 nm. Their areas were utilized to quantify monomeric and oligomeric species. As summarized in Table 2, the fraction of monomer in the same purification conditions is generally higher for MBP-16E6mut than for MBP-8E6. This result confirms that MBP-8E6 is highly prone to self-association, consistent with our previous observations.

**Table 2:** Summary table for purification results: Monomer are defined based on their elution volume, as it follows: MBP-16E6mut: V_e_ = 1.92 ± 0.01 mL; MBP-8E6: V_e_ = 1.99 ± 0.04 mL Oligomers are defined as all protein species eluted between void volume (V_0_) (included) and monomer elution volume (excluded)

	Monomer (%)	Oligomer (%)	Total A280 GF peak area (mAU × mL)
A: MBP-16E6mut			
(1) Filter elution			
N1	95	5	256
N2	87	13	243
N3	52	48	167
N4	85	15	480
N5	30	70	116
N6	68	32	345
A	10	90	263
(2) Pipetting elution			
N1	86	14	366
N2	88	12	125
N3	60	40	125
N4	72	28	128
N5	39	61	39
N6	69	31	155
B: MBP-8E6			
(1) Filter elution			
N1	19	81	467
N2	30	70	113
N3	12	88	230
N4	22	78	256
N5	7	93	194
N6	9	91	404
A	16	84	486
(2) Pipetting elution			
N1	71	29	301
N2	56	44	117
N3	28	72	227
N4	56	44	121
N5	16	84	156
N6	74	26	89
(3) Optimization of lysate incubation time			
N1			
5 min	74	26	185
30 min	77	23	160
2 h	56	44	220
N2			
5 min	92	8	25
30 min	71	29	38
2 h	77	23	138

Quantification of monomeric and oligomeric species is based on the integration of elution peak (absorbance at 280 nm), with estimated uncertainty of ± 15% for monomer/oligomer ratio and ± 57 mAU × mL for total A80 peak area based on duplicated experiments

**Fig. 4:**
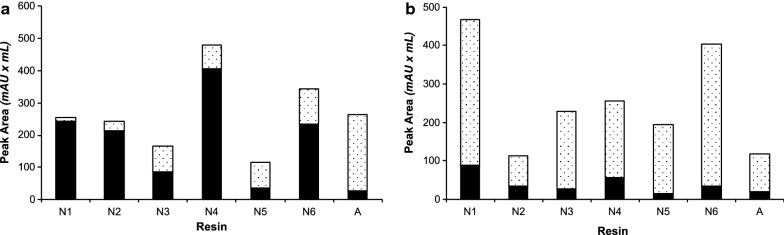
Tests of various reticulated affinity resins (N1–6: Nickel resins; A: amylose resin, as detailed in Table 1). Elution was recovered by centrifugal filtration (5 min at 500×*g*) of the elution buffer–resin mixture. The ratio between monomer and total protein amount [monomer (black bars) + oligomers (dotted bar)] were calculated from the peak areas of the corresponding elution volumes. **a** MBP-16E6mut. **b** MBP-8E6

The difference between the MBP-16E6mut reference protein and the challenging MBP-8E6 protein is particularly visible in Fig. 4, since the proportion of oligomers over total purified protein is much higher for MBP-8E6 (values comprised between 70 and 93% for the different tested nickel resins, Table 2) than for MBP-16E6mut (between 5 and 70%). It also clearly appears that amylose resin retains a higher proportion (90%) of MBP-16E6mut oligomers than nickel resins, confirming the hypothesis of oligomer exclusion by reticulated nickel resins. A standard deviation of 15% unit was inferred from the duplication of several experiments, warranting significance. Three nickel resins allow to purify 85% or more of monomeric MBP-16E6mut, namely N1 (95%), N2 (87%) and N4 (85%). In addition, quantification of the total protein amount (Table 2, see “Total A280 GF Peak Area”) shows that N4 resin has the highest recovery capacity of total purified protein. Regarding the purified MBP-8E6 sample, the same three resins N1, N2 and N4 show the maximal ratio of monomeric state (19%, 30% and 22%, respectively), although the monomer fraction obtained with the amylose resin (16%) is within the same range as that obtained with nickel resins (7 to 30%).

The present assays showed the usefulness of using a reticulated nickel resin for the purification of MBP-16E6mut, since purification trials allowed to reach up to 95% monomer in a single purification step. However, MBP-8E6, being more prone to oligomerization, could not yet be purified as a predominantly monomeric state at this step.

### Optimizing the elution protocol to maximize the proportion of monomeric protein

At the previous stage, resins were thoroughly dried by centrifugal filtration in order to recover maximal liquid fraction and to quantify all oligomer species that were bound (filter elution protocol). After probing the efficiency of nickel reticulated resins, we implemented another elution method aimed at reducing the fraction of oligomers in the final purified fraction. Instead of being filtrated, the resin/elution buffer mixture was centrifuged at low speed (5 min at 500×*g*) and the supernatant was collected immediately after centrifugation. The oligomers are expected to diffuse out of the resin slower than the monomers, thereby increasing the proportion of monomers in the final pipetted material.

**Fig. 5:**
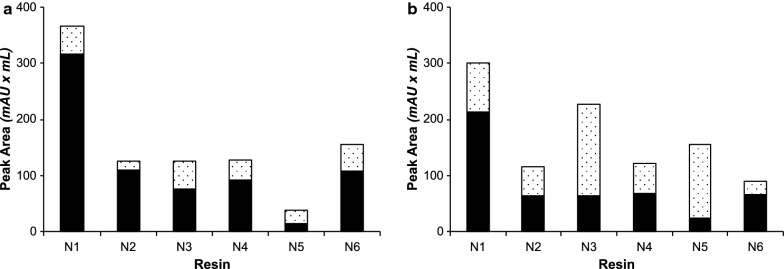
Test of the “decant and take up” elution method on nickel resins (N1–6: Nickel; A: amylose, as detailed in Table 1). Elution was recovered by pipetting the supernatant after gentle centrifugation (5 min at 500×*g*) of the elution buffer–resin mixture. Monomer: black bars; oligomers: dotted bars. **a** MBP-16E6mut. **b** MBP-8E6

Using this adapted elution protocol, the proportion of monomeric MBP-8E6 was significantly improved and reached up to 71% for resin N1 (Fig. 5). Resins N2 and N4 allowed to purify up to 56% of monomeric protein, but N1 remains the only resin allowing to purify the highest total protein concentration and the largest monomer ratio (as defined by the proportion of monomer over the total amount of protein in the sample) for both MBP-8E6 and MBP-16E6mut samples. For MBP-16E6mut, the two resins reaching highest monomer ratio with the new elution method are N1 and N2, with approximately 86% and 88%, respectively. Although the monomer fraction was significantly increased with this “decant and take up” elution procedure, it is noticeable that the total protein amount was reduced by two-fold for most of the resins. By adapting the elution method, we have shown that it is possible to significantly reduce the diffusion of oligomeric species in solution, and subsequently to maximize the ratio of monomeric purified protein.

### Reducing the contact time of bacterial extract with resin during the initial binding step limits protein oligomer binding

Contact time between affinity resin and cleared bacterial lysate during the initial binding step is another critical parameter for protein purification since it determines the amount and the nature of protein species bound to the resin. On the one hand, a long incubation time should increase the total amount of protein that will be recovered. On the other hand, a shorter incubation time might restrict the size of the proteins interacting with the resin. As large particles such as soluble oligomers diffuse slower in solution, their binding on affinity resin should thus be limited when incubation time is short.

**Fig. 6:**
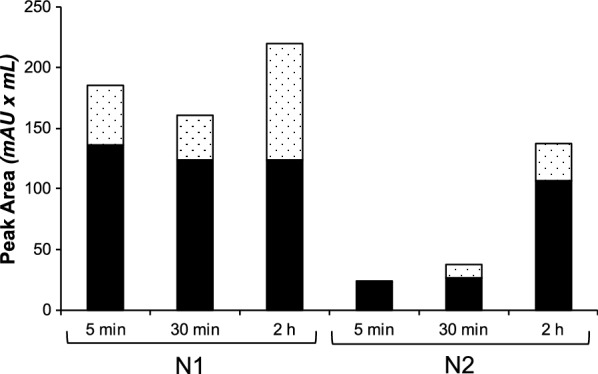
Optimization of bacterial extract contact time for the purification of MBP-8E6 on nickel resins N1 and N2: three contact times were tested (5 min; 30 min; 2 h). Monomer: black bars; oligomers: dotted bars

To assess this hypothesis, we selected two resins showing the highest monomer ratio for both the reference MBP-16E6mut and the challenging MBP-8E6 proteins, namely N1 and N2. Since the “decant and take up” elution method already improved the fraction of monomeric MBP-16E6mut by up to 70–90%, this third step in the optimization protocol was focused on MBP-8E6, the protein most prone to self-association in our study. Incubation time was set at either 30 min or 5 min and systematically compared to the 2-h reference time used in the previous trials. Elution was done by pipetting the supernatant after low-speed centrifugation of the resin resuspended in elution buffer. The results presented in Fig. 6 show that the shorter the contact time between the cleared lysate and the resin, the higher the proportion of monomer in the final purified fraction, confirming our assumption. With the N1 resin, about 75% of monomers were purified with either 30 min or 5 min contact time whereas this ratio dropped down to 56% for the 2-h incubation delay. The best monomer ratio for monomeric MBP-8E6 (92%) was obtained by using the N2 resin for purification, with a 5 min contact time and the “decant and take up” elution method. Considering that only 30% of monomers were obtained using this same resin and applying the initial protocol (i.e. 2-h contact time and elution by filtration), this result shows that our optimization brought a significant improvement to purify a biochemically problematic HPV E6 protein. One should note that this quality refinement of the purified protein is at the expense of a decrease of the total amount of the MBP-8E6 protein. It is particularly visible from the purifications with the N2 resin, for which a fivefold decrease of the protein quantity was observed between 2-h and 5-min contact time.

**Fig. 7:**
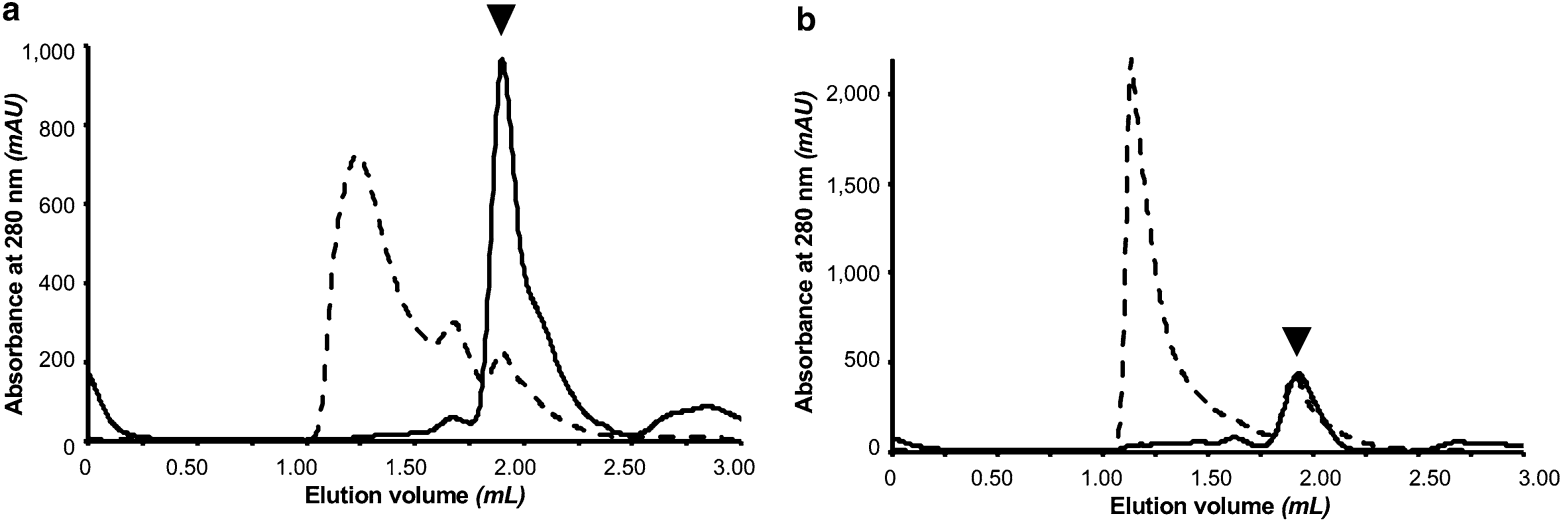
SEC chromatograms for purified MBP-E6 proteins with initial protocol, represented with dashed line (amylose resin; 2 h contact time between bacterial lysate and resin; filter elution) versus optimized protocol, represented with continuous line (N2 nickel resin; 5 min contact time; “decant and take up” elution). The elution peak corresponding to monomeric protein is indicated by the arrow. **a** MBP-16E6mut. **b** MBP-8E6

For MBP-16E6mut, the absolute amount of monomeric protein was further increased by applying the above refined protocol (Fig. 7). In contrast, the amount of monomeric MBP-8E6 remained approximately the same with both purification strategies. The increase of the ratio of monomeric protein with the optimized protocol can be explained by the drastic elimination of oligomeric species (in particular those from the void volume at 1.18 mL).

### Probing the activity of the purified protein by SPR

In order to assess the activity of protein samples purified according to the optimized protocol, we performed a protein–peptide interaction assay by SPR. This was achieved using 16E6mut in order to compare with SPR data previously obtained on a related 16E6 mutant using the original large-scale approach [30].

We purified MBP-16E6mut according to our batch protocol: 5 min contact time between the bacterial lysate and the affinity resin, followed by “decant and take up” elution by pipetting the supernatant after short centrifugation in Eppendorf tube. We performed this protocol with N1 and N2, the two resins that gave the highest monomer ratio after purification with the optimized elution protocol. In order to remove imidazole from protein solution, the final fractions were desalted in the SPR running buffer by bench desalting column.

Following this protocol, we purified the equivalent of 8.5 µmol of MBP-16E6mut per liter of bacterial culture, which is largely sufficient to perform parallel SPR assays (in our setup, the minimum protein amount required for one SPR assay including 10 cycles is 2 nmole).

**Fig. 8:**
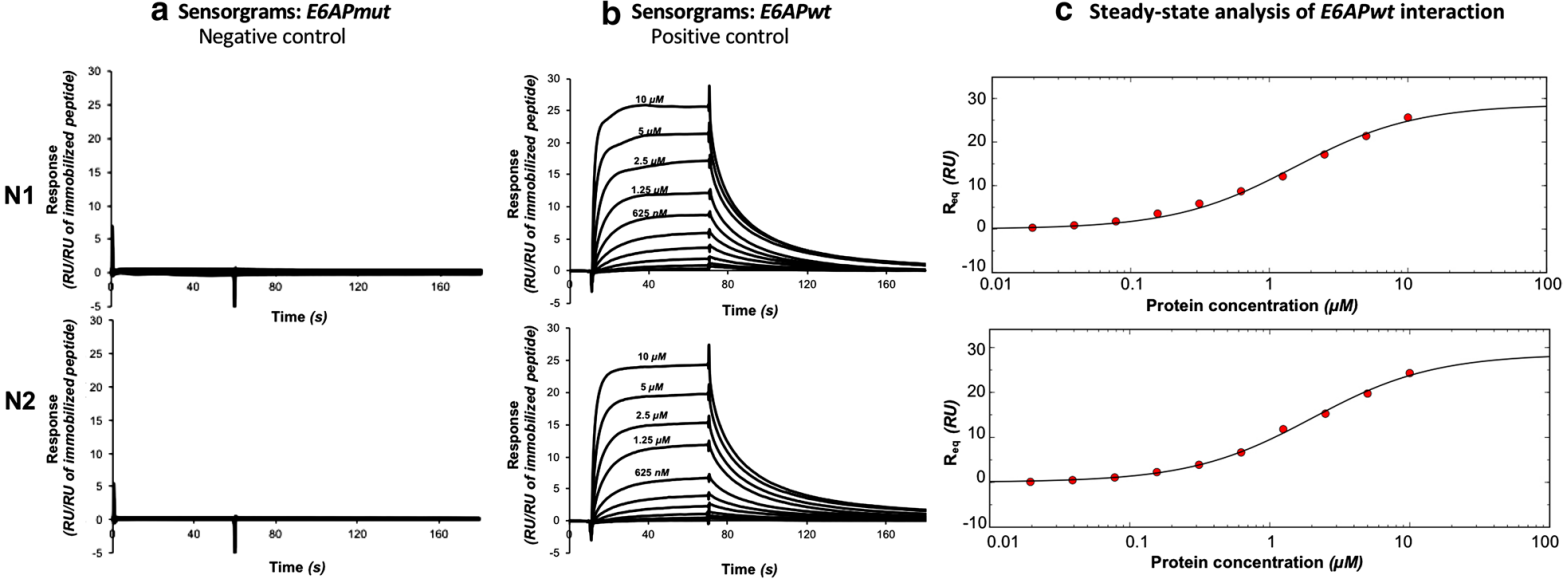
SPR data for the assessment of MBP-16E6mut interaction properties. The protein was purified on nickel resins N1 (top) and N2 (bottom) according to the optimized protocol (5 min contact time between the bacterial extract and the affinity resin, “decant and take up” elution). **a** Sensorgrams for the interaction with E6APmut, plotting the response normalized by the peptide immobilization level versus time. **b** Sensorgrams for the interaction with peptide E6APwt, same axes. **c** Steady-state analysis of interaction with E6APwt, plotting the equilibrium response R_eq_ obtained in a 5-s. Window versus protein concentration

MBP-E6 was tested against its prototypical target peptide, the 16-mer LXXLL motif of E6AP (PESSELTLQELLGEER, called thereafter *E6APwt*) [21]. The CAPture kit system (GE Healthcare) was used to reversibly immobilize biotinylated peptides on the chip. Each cycle consists of an immobilization of the peptide, followed by the injection of the protein at a given concentration, and a regeneration step to wash out both the remaining protein and the immobilized peptide of the surface. Figure 8b shows the superimposition of time-aligned sensorgrams obtained for different concentrations after reference substraction and normalization according to the peptide immobilization level. As a negative control, we tested the interaction against an E6AP mutant (PESSELT**A**QELLGEER, called thereafter *E6APmut*) abolishing the binding (Fig. 8a). No significant signal was observed for this control. In particular, the association phase does not display any concentration-dependent signal.

Steady-state analysis was performed to estimate dissociation constants (K_D_) of the MBP-16E6mut interacting with *E6APwt* (Fig. 8c). For the protein samples purified on resins N1 and N2, the estimated K_D_ were 1.6 ± 0.3 µM and 2.0 ± 0.3 µM, respectively. These data are in close agreement with a previously published SPR analysis of the binding of purified HPV16 E6 to the LXXLL motif of E6AP [30]. In that study, the obtained K_D_ was 4.15 ± 0.52 µM. The moderate difference in the absolute affinity constant measured in that previous work might be due to several changes in the experimental setup. In the former publication, the recombinant peptide was fused to GST and further captured on CM5 chip via an anti-GST antibody, whereas biotinylated synthetic peptides used in the present study were reversibly captured on CAP chip. In addition, the previous publication used a distinct mutant of HPV16 E6, called E6 6C/6S. This former mutant included two additional cys/ser mutations that were later shown to slightly destabilize the N-terminal domain of HPV16 E6 [31]. Despite these experimental differences, the good agreement between affinity measurements indicates that the optimized single-step purification protocol is efficient to obtain protein sample in an active form.

## Discussion

Quality of recombinant protein sample can be improved by optimizing either the expression or the purification process. The present work mainly focused on the purification process to maximize the yield of soluble overexpressed protein. Our purpose was to obtain an MBP-E6 sample as homogeneous as possible from the available material based on the expression conditions in *E. coli* that we previously published [9].

E6 oncoproteins from papillomaviruses contain two zinc-binding domains and have been found to interact with some host proteins through LXXLL motif recognition, such as E6AP ubiquitin ligase [32]. After overexpression in *E. coli* and purification, it was reported that MBP-E6 forms mixtures of soluble active monomers and soluble oligomers comprising misfolded E6 moieties (thus unable to specifically bind LXXLL motifs) [10]. These oligomers can be eliminated by overnight ultracentrifugation followed by size-exclusion chromatography [22]. Although this approach is appropriate for X-ray crystallography, a faster and easier protocol was designed here for the purpose of performing protein–peptide interaction assays in parallel. As compared to structural studies, binding assays generally need less material, yet high homogeneity and solubility are required. Thus, our ultimate quality criterion was the percentage of active protein rather than the overall purification yield. We designed a single-step batch purification process maximizing the proportion of monomeric and active MBP-E6 protein. This approach is compatible with parallel purifications and generates sufficient amounts of protein for our biophysical assays. In the present study, we investigated the purification of two HPV E6 oncoproteins fused to MBP: the solubility-enhanced mutant HPV16 E6 F47R 4C/4S (16E6mut) whose structure was solved by X-ray crystallography, and the HPV8 E6 (8E6) which, in our experience, was particularly recalcitrant to purify as a monomer.

This study aimed at the preferential selection of monomeric protein species within a single purification step. Oligomers can be distinguished from monomers based on their size, more precisely their volume in solution. The radius of a particle in solution is correlated with the diffusion coefficient D according to the Stokes–Einstein formula [33]:

$$D = kT/6\pi \eta r$$

with *k* the Boltzmann constant, *T* the temperature, *η* the viscosity coefficient of the medium and *r* the radius. Thus, larger protein species diffuse slower than monomers in solution. Based on these size and diffusion properties, we optimized three parameters in the purification protocol: (i) reticulation of affinity resin, (ii) contact time between the bacterial lysate and the resin and (iii) elution by “decant and take up” method. Adapting the resin reticulation is a way to select particles based on their size in solution that is faster and easier to parallelize than SEC. The two other optimization lines rely on the distinct diffusion properties of oligomers and monomers. First, a reduced contact time limits the binding of oligomeric species on the resin. Second, a fast elution method by pipetting only the supernatant fraction enables the preferential recovery of monomeric species.

To discriminate particles based on their volume in solution, reticulated resins can be selected to set a specific exclusion limit by adapting the pore size and the agarose concentration. Protein species exceeding a threshold hydrodynamic volume in solution will not be able to enter the affinity resin pores. This property allows a double selection based on the protein size and the affinity tag, within a single step.

We compared the size of particles retained on different types of resin, amylose resin and nickel resins with distinct reticulation levels. In particular, resin N2 was a 10% cross-linked agarose resin excluding proteins above 500 kDa (10% BCL Agarose Bead Standard, ABT) that was functionalized to bind Nickel, whereas all other nickel-chelating resins have an exclusion limit of about 4000 kDa. Our results (notably with MBP-16E6mut) showed that amylose resin leads to the purification of a larger fraction of oligomers than nickel resins. N2 resin was the most efficient resin to purify the monomeric form of both MBP-16E6mut and MBP-8E6, which confirms the efficiency of adapting affinity resin pore size to limit the purification of aggregates. N1 resin was the second most efficient resin to maximize the ratio of monomeric protein. In addition, the highest total protein amount (including both monomer and oligomers) was purified with N1 resin.

The proportion of purified unwanted oligomers can be minimized by reducing incubation times at key steps of the process. First, the contact time between the bacterial crude extract and the affinity resin can be reduced in order to minimize the binding of oligomers, as demonstrated with the recalcitrant MBP-8E6 construct for which the highest monomer fraction was obtained for the minimum contact time. Second, the elution method can be designed to recover a predominantly monomeric species capable to rapidly diffuse to the supernatant. This approach increased by two-fold the ratio of purified monomeric MBP-8E6, despite its natural tendency to form a majority of oligomeric species.

Finally, we were able to confirm by SPR the binding affinity between *E6APwt* LXXLL peptide and MBP-16E6mut purified according to our optimized protocol and using the two nickel reticulated resins that allowed to purify highest proportion of monomeric protein (N1 and N2). The protein did not interact with the mutated motif *E6APmut* (AXXLL), which rules out the possibility of unspecific interactions that might happen with misfolded proteins and/or aggregated proteins.

## Conclusions

In the present work, we designed a single-step purification strategy optimized for maximal recovery of monomeric MBP-E6 that we accurately quantified by means of analytical SEC. By using a customized reticulated resin, adapting the contact time and performing fast elution, we were able to reach high monomer ratios in the final purified fraction. This method was particularly efficient for the solubility-enhanced mutant MBP-16E6mut and the highly aggregation-prone MBP-8E6. As assessed by SPR, the purified MBP-16E6mut protein interacts with its known partner within the expected range of affinity. This method is promising for forthcoming biophysical studies with recalcitrant proteins, while allowing to perform parallel purifications. The approach should also be easily adaptable to robotization. It would be interesting to automatize this protocol for purifying at high throughput families of proteins, which, like E6 oncoproteins, are difficult to fold except when fused to MBP or other soluble carrier proteins. The overexpression of such constructs leads to a limited proportion of properly folded and active monomers that require to be separated from non-active oligomers, which is now possible using the protocol presented here.

## Methods

### Customized Nickel resin N2

#### Preparation

High Density Nickel 10BCL has been manufactured as a customized product by Agarose Bead Technologies. The resin consists of crosslinked 10% agarose beads to which a Nickel chelating group has been immobilized. This chelating group has been obtained by a modification of the procedures described elsewhere [34, 35] using 10% BCL Agarose Bead Standard as raw material. The epoxide generated has been treated with iminodiacetic acid and charged with Ni^2+^ ions according to a modification of the method previously described.

#### Characterization

The characteristics of 10% BCL Agarose Bead Standard for protein separation based on their molecular weight are the following: the fractionation range for globular proteins is 1 × 10^4^–5 × 10^5^ Da and the exclusion limit is > 5 × 10^5^ Da. Nickel was quantified by spectrophotometric assay, resulting in 34 µmol Ni^2+^/mL gel.

### Expression and fast batch purification procedure

The sequences encoding for 16E6mut (already described in [22]) and for 8E6 were cloned into a pETM-41 vector by using NcoI and Acc65I sites, allowing the production of MBP-6His-TEV site-E6 fusions (Fig. 1). The sequence encoding for 8E6 was kindly provided by Yves Jacob from Pasteur Institute (Paris, France) and corresponds to a variant with three substitutions as compared to the reference protein sequence (Uniprot ID: P06428), namely Y9N, A26E and S36L.

Both constructs were expressed in *Escherichia coli* BL21 (DE3) cells, grown in LB medium supplemented with kanamycine 50 µg/mL at 37 °C until OD_600_ ≈ 0.7. Expression was induced by adding 0.5 mM IPTG (Isopropyl β-d-1-thiogalactopyranoside) and 100 µM ZnSO_4_. Cells were grown overnight at 16 °C and harvested by centrifugation. Pellets were stored at − 20 °C.

Purification buffers were thoroughly degassed and equilibrated with argon before adding 2 mM DTT. Culture pellets were then resuspended in lysis buffer, which was Buffer A (Tris 50 mM pH 8; NaCl 400 mM; DTT 2 mM) supplemented with 5% (w/v) glycerol, 0.25 µg/mL RNase I, 0.25 µg/mL DNase I, lysozyme at approximately 1 µg/mL and anti-protease cocktail EDTA-free (Roche) according to the manufacturer instruction. This buffer was supplemented with 10 mM imidazole for purification on Nickel resins. Pellet equivalent to 250 mL culture volume was resuspended in 10 mL lysis buffer. Cells were lysed by sonication on ice and then centrifuged at 100,000×*g* at 4 °C for 45 min. The supernatant was then incubated at 4 °C with either nickel (100 µL resin for 250 mL culture pellet) or amylose resins (300 µL resin for 250 mL culture pellet), equilibrated in Buffer A supplemented with 20% of the recommended concentration of anti-protease cocktail. The incubation time of the cleared lysate with the resin was 2 h in the initial purification tests, before to be evaluated as a variable parameter (5 min, 30 min or 2 h) and reduced to 5 min in the final optimized protocol. The resins were pelleted by 5 min-centrifugation at 500×*g* at 4 °C; the supernatant was discarded and the resins were first washed in Buffer A (30 min incubation at 4 °C), then in Buffer B (Tris 50 mM pH 8, NaCl 1 M, DTT 2 mM, 20% of the recommended concentration of anti-protease cocktail and supplemented with 10 mM imidazole for Nickel resins) with same incubation time and temperature. Resins were re-equilibrated in Buffer A during 10 min at 4 °C before elution. Resins were then transferred in Eppendorf tubes, pelleted by centrifugation for 5 min at 500×*g*, 4 °C and supernatants were discarded. Finally, resins were resuspended in elution buffer (Buffer A supplemented with 20% anti-protease cocktail and, for Nickel resins: imidazole 600 mM; for Amylose resin: maltose 15 mM). 100 µL Nickel resin were eluted with 140 µL imidazole elution buffer while 300 µL amylose resin were eluted with 200 µL maltose elution buffer.

For standard elution, resins were transferred in 96-well filter plate and centrifuged 5 min at 500×*g* and 4 °C for maximal recovery of liquid phase by filtration. For the optimized elution protocol, the tube content was then rapidly mixed by gentle vortex and immediately centrifuged during 5 min at 500×*g* and 4 °C. The supernatant was recovered and used as final purified protein solution. Protein concentration was determined by Nanodrop measurement, based on absorbance at 280 nm. Final protein samples were systematically analyzed either by SDS-PAGE or by microfluidics capillary gel electrophoresis in order to check the presence of protein contaminants in denaturing conditions. Prior to SPR assays, protein samples were desalted on Illustra NAP-5 columns (GE Healthcare) equilibrated in Buffer A allowing to eliminate imidazole or maltose from the solution.

### Microfluidics capillary gel electrophoresis

Purification samples (from the washing steps and elution) were transferred in 96-well plates, mixed with the Caliper sample buffer and boiled according to the manufacturer instructions. The plates were loaded and measured on LabChip GX II device (Caliper, Perkin Elmer), with HT Protein Express 100 High Sensitivity protocol (10–100 kDa). The kit allows to label the proteins with a fluorescent dye. This dye is excited by a laser during protein capillary electrophoresis in denaturing conditions. The emitted fluorescence signal is plotted versus migration time, leading to the protein separation according to their molecular weight. Data processing is performed with LabChip GX II software. Migration time is first converted into molecular weight using standard protein markers before a quantitative analysis is performed thanks to empirical linear correlation between fluorescence intensity and protein amount.

### Analytical size-exclusion chromatography

Samples used for analytical SEC were centrifuged 5 min at 13,700×*g*, 4 °C and only the supernatant was loaded on the column Superdex 200 Increase 5/150 GL (GE Healthcare) previously equilibrated in Buffer A. SEC run was performed on ÄKTA purifier (GE Healthcare), by injecting 50 µL per sample, with flow set at 0.150 mL/min. Spikes of absorbance 280 nm signal due to air bubbles were excluded from the chromatogram before performing data analysis. The original chromatograms are provided in Additional file 2.

We calibrated the column with Gel Filtration Calibration Kit High Molecular Weight (GE Healthcare), using a solution of dextran Blue (0.1 mg/mL) and two mixes. Mix 1 was composed of ovoalbumine (4 mg/mL), aldolase (4 mg/mL) and thyroglobuline (5 mg/mL). Mix 2 contained ferritine (0.3 mg/mL) and conalbumine (3 mg/mL). Each of the three calibration solution was loaded in a 50 µL loop and injected separately at a flow rate of 0.150 mL/min on the column previously equilibrated in Buffer A. The resulting elution volumes were used to establish a calibration plot to determine the molecular weight *MW*, based on the following equation [33]:

$$K_{av} = \frac{{V_{e} - V_{o} }}{{V_{t} - V_{o} }}$$

where *K*_*av*_ stands for the available distribution coefficient, *V*_*e*_ corresponds to the elution volume of each injected protein, *V*_*o*_ is the void volume of the column (determined by the elution volume of the dextran blue) and *V*_*t*_ is the total bed volume (3 mL with this specific column). Thus, the coefficients *a* and *b* were determined by linear regression using the following equation [33]:

1 $$K_{av} = a \times \log \left( {MW} \right) + b$$

Moreover, the hydrodynamic radius *R*_*h*_ was determined according to:

2 $$\sqrt { - \log_{10} \left( {K_{av} } \right)} = c \times R_{h} + d$$

where *c* and *d* coefficients are determined by linear regression [33].

The area of elution peaks (based on absorbance at 280 nm) was calculated using the evaluation module of Unicorn 5.31 software.

According to the column calibration, we inferred the elution volume of monomeric (approximately 1.92 mL) and oligomeric MBP-E6 (V_e_ < 1.90 mL). For each assay, the area of the elution peak corresponding to the monomer was divided by the area of peaks corresponding to all eluted protein species (monomeric and oligomeric), allowing us to measure the ratio of monomer and oligomer in the purified protein sample.

Regarding the ratios of monomeric protein, we observed that the duplication of the data leads to standard deviations never exceeding 15% unit. This value has consequently been considered as the uncertainty of monomer/oligomer ratios in this work. This uncertainty, although given as a percentage, corresponds to the absolute variation of the ratio of monomeric protein and not as a relative variation coefficient.

### Peptide synthesis

To test the ability of the purified protein to interact with LXXLL peptides, we used two synthetic peptides: *E6APwt* (PESSELTLQELLGEER) and *E6APmut* (PESSELTAQELLGEER). Both peptides were N-ter biotinylated with a TTDS spacer (*N*-(13-amino-4,7,10-trioxa-tridecayl)-succinamic acid) (Iris Biotech GMBH) and synthesized either by JPT Innovative Peptide Solutions or by the peptide synthesis service at IGBMC with 70–80% purity. The lyophilized peptides were resuspended in water at a final stock concentration of 5 mM and stored at − 20 °C.

### Surface plasmon resonance

Peptide–protein interaction assays were performed by surface plasmon resonance (SPR) on a Biacore T200 instrument (GE Healthcare—Biacore) at 25 °C. The running buffer was Tris 50 mM pH 8, NaCl 400 mM, DTT 2 mM and 0.005% (v/v) surfactant polysorbate 20 (GE Healthcare). We used Biotin CAPture kit (GE Healthcare—Biacore) for the reversible capture of biotinylated peptides on a chip. Briefly, the chip is coated with a deoxyribooligonucleotide that hybridizes with the complementary strand bound to streptavidin. The biotinylated peptide binds to streptavidin and can be washed by dehybridizing the two oligonucleotides. An empty control surface was systematically included on every cycle to serve as a reference for non-specific binding of the analyte to the matrix and for monitoring changes in solution refractive index. This reference surface was treated as the peptide surfaces except that peptide injection was omitted. Kinetic runs were performed by injecting series of two-fold cascade dilutions of the analyte MBP-E6 samples, starting from 10 µM.

Thus, at the beginning of each binding cycle, CAPture reagent (diluted 5 times in running buffer) was injected on all channels during 300 s at 2 µL/min. For peptide surfaces, peptide solution (50 nM) was injected during 15–30 s at 10 µL/min in order to reach 4–15 RU immobilization level. Protein sample was then injected during 60 s at 30 µL/min, followed by 120 s buffer flow. The surfaces were then regenerated by injecting regeneration solution as indicated by the manufacturer (guanidine hydrochloride 6 M; sodium hydroxide 250 mM) for 60 s at 5 µL/min.

The SPR signals from the regions corresponding to the protein injection and post-injection phases were plotted as RU versus time. Data were first processed using the Biacore T200 Evaluation 1.0 software (GE Healthcare, Biacore Life Science, Uppsala, Sweden). Sensorgrams obtained for the different protein concentrations were corrected for buffer effects and bulk refractive index changes by subtracting the empty cell signal, and subsequently normalized according to the peptide levels which can slightly differ from cycle to cycle due to the immobilization process. The steady-state binding signal was derived by averaging the signals at equilibrium within a five second-window (R_eq_). Steady-state analysis was performed using an in-house Python script by fitting the average and normalized signal R_eq_ as a function of total analyte concentration, and assuming a simple 1:1 interaction binding isotherm model. Uncertainties of the derived parameters were estimated using Monte-Carlo approach, considering an experimental uncertainty of 5 RU. This value was estimated by duplicating the full experimental cycle corresponding to a single protein concentration.

## Additional files

**Additional file 1.** Corrected size-exclusion chromatograms. Prior to protein peak integration, spikes due to air bubbles were excluded. **A**: Chromatograms for analytical SEC of MBP-16E6mut. In order to control to oligomeric state of the purified protein, we performed systematic analytical SEC on the final protein sample. The calibration of the column allowed us to estimate the size of the particles at different elution volumes, thus the elution peak of monomeric protein is indicated by an arrow. Note the increase of the monomer fraction compared to the total amount of purified protein when shifting from the filter elution to the “decant and take up” elution method. **B**: Chromatograms for analytical SEC of MBP-8E6. On this protein challenging to purify, the increase of the monomer fraction (indicated by an arrow) between the two elution methods is dramatic. **C**: Chromatograms for analytical SEC of MBP-8E6: optimization of bacterial extract contact time. The elution peak of monomeric protein is indicated by an arrow. The decrease of the oligomeric fraction (eluted between the void volume V_0_ and the monomer fraction) is particularly visible for nickel resin N1.

**Additional file 2.** Raw size-exclusion chromatograms. We provide in this file the raw data of size-exclusion chromatograms used in the study (absorbance at 280 nm versus elution volume). The protein (MBP-16E6mut, MBP-8E6), resin (N1…N6, A), elution method (filter, decant) and contact time for optimization assays (2 h, 30 min, 5 min) are indicated in the worksheet names. The first worksheet entitled Readme contains explanations on the nomenclature used for worksheet titles.

## Abbreviations

DTT: dithiothreitol
HPV: human papillomavirus
IMAC: immobilized-metal affinity chromatography
IPTG: isopropyl β-d-1-thiogalactopyranoside
MBP: maltose-binding protein
SEC: size-exclusion chromatography
SPR: surface plasmon resonance

## References

[1] Waugh DS (2016). The remarkable solubility-enhancing power of *Escherichia coli* maltose-binding protein. Postepy Biochem.

[2] Berrow NS, Büssow K, Coutard B, Diprose J, Ekberg M, Folkers GE (2006). Recombinant protein expression and solubility screening in *Escherichia coli*: a comparative study. Acta Crystallogr D Biol Crystallogr.

[3] Riggs P (2000). Expression and purification of recombinant proteins by fusion to maltose-binding protein. Mol Biotechnol.

[4] Riggs P, Ausubel FM, Brent R, Kingston RE, Moore DD, Seidman JG, Smith JA (2001). Expression and purification of maltose-binding protein fusions. Current protocols in molecular biology.

[5] Sachdev D, Chirgwin JM, Thorner J, Emr SD, Abelson JN (2000). [20] Fusions to maltose-binding protein: control of folding and solubility in protein purification. Applications of chimeric genes and hybrid proteins part A: gene expression and protein purification.

[6] Sachdev D, Chirgwin JM (1999). Properties of soluble fusions between mammalian aspartic proteinases and bacterial maltose-binding protein. J Protein Chem.

[7] Sachdev D, Chirgwin JM (1998). Order of fusions between bacterial and mammalian proteins can determine solubility in *Escherichia coli*. Biochem Biophys Res Commun.

[8] Sachdev D, Chirgwin JM (1998). Solubility of proteins isolated from inclusion bodies is enhanced by fusion to maltose-binding protein or thioredoxin. Protein Expr Purif.

[9] Nominé Y, Ristriani T, Laurent C, Lefèvre J-F, Weiss É, Travé G (2001). A strategy for optimizing the monodispersity of fusion proteins: application to purification of recombinant HPV E6 oncoprotein. Protein Eng.

[10] Nominé Y, Ristriani T, Laurent C, Lefèvre J-F, Weiss É, Travé G (2001). Formation of soluble inclusion bodies by HPV E6 oncoprotein fused to maltose-binding protein. Protein Expr Purif.

[11] Zanier K, Nominé Y, Charbonnier S, Ruhlmann C, Schultz P, Schweizer J (2007). Formation of well-defined soluble aggregates upon fusion to MBP is a generic property of E6 proteins from various human papillomavirus species. Protein Expr Purif.

[12] Zanier K, Ruhlmann C, Melin F, Masson M, Ould M’hamed Ould Sidi AO, Bernard X (2010). E6 proteins from diverse papillomaviruses self-associate both in vitro and in vivo. J Mol Biol.

[13] Raran-Kurussi S, Waugh DS (2012). The ability to enhance the solubility of its fusion partners is an intrinsic property of maltose-binding protein but their folding is either spontaneous or chaperone-mediated. PLoS ONE.

[14] de Marco A (2013). Recombinant polypeptide production in *E. coli*: towards a rational approach to improve the yields of functional proteins. Microb Cell Fact..

[15] de Marco A, Lorence A (2012). Optimization of purification protocols based on the step-by-step monitoring of the protein aggregates in soluble fractions. Recombinant gene expression.

[16] Suarez I, Trave G (2018). Structural insights in multifunctional papillomavirus oncoproteins. Viruses.

[17] Wallace NA, Galloway DA (2015). Novel functions of the human papillomavirus E6 oncoproteins. Annu Rev Virol.

[18] Vande Pol SB, Klingelhutz AJ (2013). Papillomavirus E6 oncoproteins. Virology.

[19] Ould M’hamed Ould Sidi A, Ould Babah K, Brimer N, Nominé Y, Romier C, Kieffer B (2011). Strategies for bacterial expression of protein–peptide complexes: application to solubilization of papillomavirus E6. Protein Expr Purif.

[20] White EA, Howley PM (2013). Proteomic approaches to the study of papillomavirus–host interactions. Virology.

[21] Scheffner M, Huibregtse JM, Vierstra RD, Howley PM (1993). The HPV-16 E6 and E6-AP complex functions as a ubiquitin–protein ligase in the ubiquitination of p53. Cell.

[22] Zanier K, Charbonnier S, Sidi AOMO, McEwen AG, Ferrario MG, Poussin-Courmontagne P (2013). Structural basis for hijacking of cellular LxxLL motifs by papillomavirus E6 oncoproteins. Science.

[23] Martinez-Zapien D, Ruiz FX, Poirson J, Mitschler A, Ramirez J, Forster A (2016). Structure of the E6/E6AP/p53 complex required for HPV-mediated degradation of p53. Nature.

[24] Tommasino M (2014). The human papillomavirus family and its role in carcinogenesis. Semin Cancer Biol.

[25] Bzhalava D, Eklund C, Dillner J (2015). International standardization and classification of human papillomavirus types. Virology.

[26] Van Doorslaer K, Tan Q, Xirasagar S, Bandaru S, Gopalan V, Mohamoud Y (2012). The Papillomavirus Episteme: a central resource for papillomavirus sequence data and analysis. Nucleic Acids Res.

[27] de Sanjose S, Quint WG, Alemany L, Geraets DT, Klaustermeier JE, Lloveras B (2010). Human papillomavirus genotype attribution in invasive cervical cancer: a retrospective cross-sectional worldwide study. Lancet Oncol.

[28] Gillison ML, Alemany L, Snijders PJF, Chaturvedi A, Steinberg BM, Schwartz S (2012). Human papillomavirus and diseases of the upper airway: head and neck cancer and respiratory papillomatosis. Vaccine.

[29] Tommasino M (2017). The biology of beta human papillomaviruses. Virus Res.

[30] Zanier K, Charbonnier S, Baltzinger M, Nominé Y, Altschuh D, Travé G (2005). Kinetic analysis of the interactions of human papillomavirus E6 oncoproteins with the ubiquitin ligase E6AP using surface plasmon resonance. J Mol Biol.

[31] Zanier K, Ould M’hamed Ould Sidi A, Boulade-Ladame C, Rybin V, Chappelle A, Atkinson A (2012). Solution structure analysis of the HPV16 E6 oncoprotein reveals a self-association mechanism required for E6-mediated degradation of p53. Structure.

[32] Brimer N, Drews CM, Vande Pol SB (2017). Association of papillomavirus E6 proteins with either MAML1 or E6AP clusters E6 proteins by structure, function, and evolutionary relatedness. PLoS Pathog.

[33] Tayyab S, Qamar S, Islam M (1991). Size exclusion chromatography and size exclusion HPLC of proteins. Biochem Educ.

[34] Hubert P, Porath J (1980). Metal chelate affinity chromatography: I. Influence of various parameters on the retention of nucleotides and related compounds. J Chromatogr A.

[35] Sundberg L, Porath J (1974). Preparation of adsorbents for biospecific affinity chromatography: I. Attachment of group-containing ligands to insoluble polymers by means of bifunctional oxiranes. J Chromatogr A.

