# Supplementary material for: One-step affinity purification of fusion proteins with optimal monodispersity and biological activity: application to aggregation-prone HPV E6 proteins
Source: Microb Cell Fact. 2018 Dec 1;17:191. doi: 10.1186/s12934-018-1039-z (PMC6271572; doi:10.1186/s12934-018-1039-z)

A. Chromatograms for analytical SEC of 16E6mut

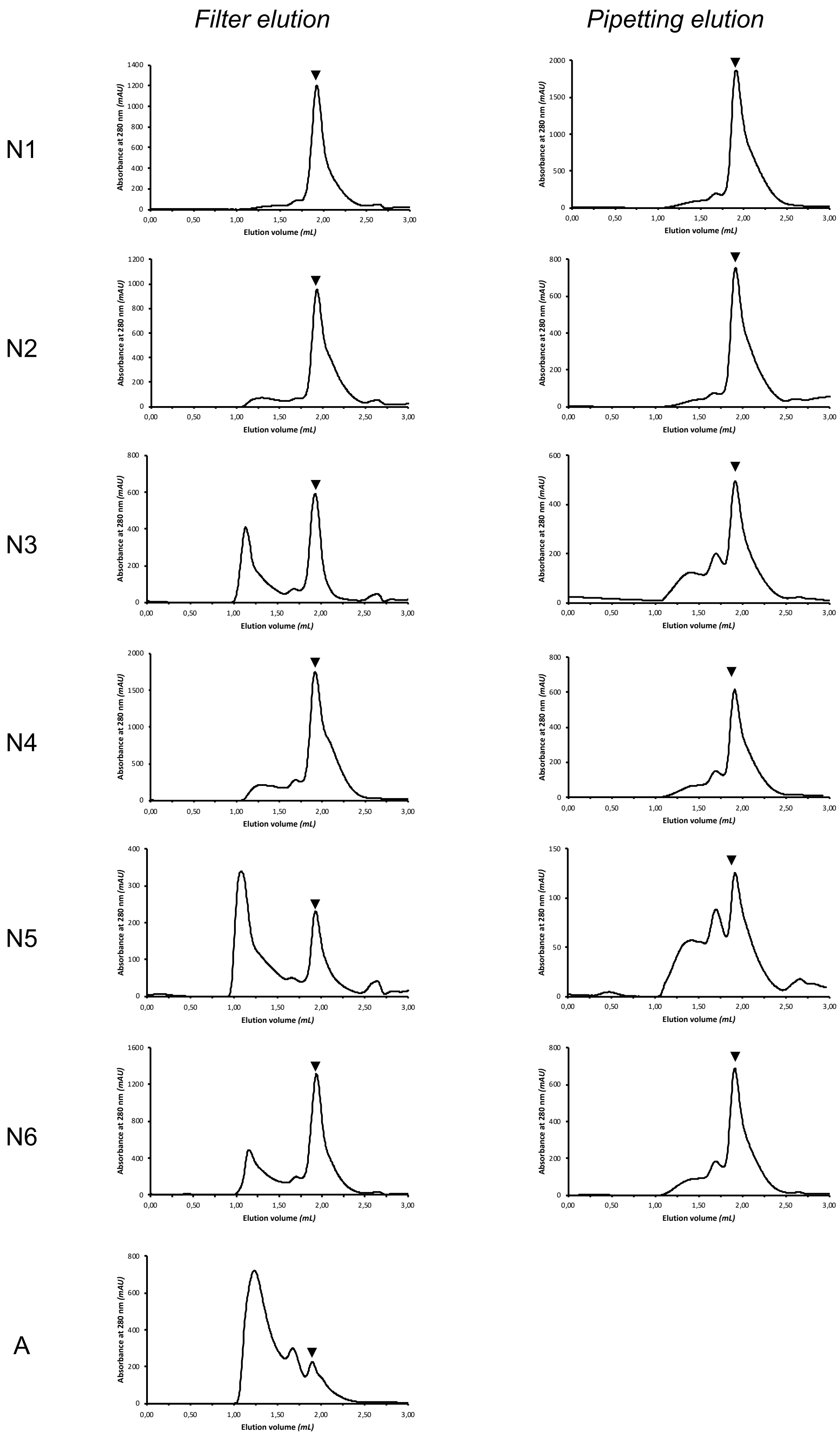

B. Chromatograms for analytical SEC of 8E6

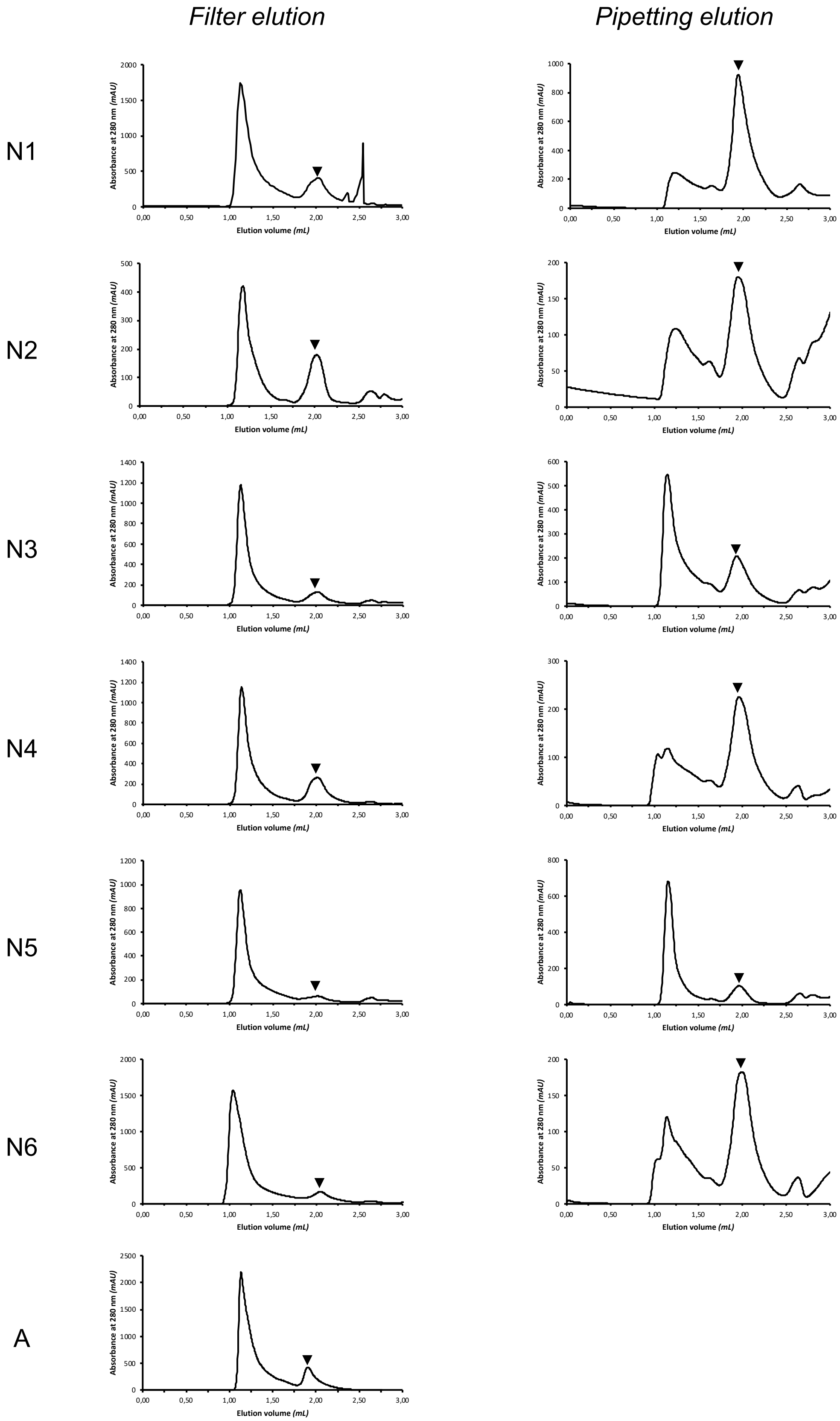

C. Chromatograms for analytical SEC of 8E6:
Optimization of bacterial extract contact time

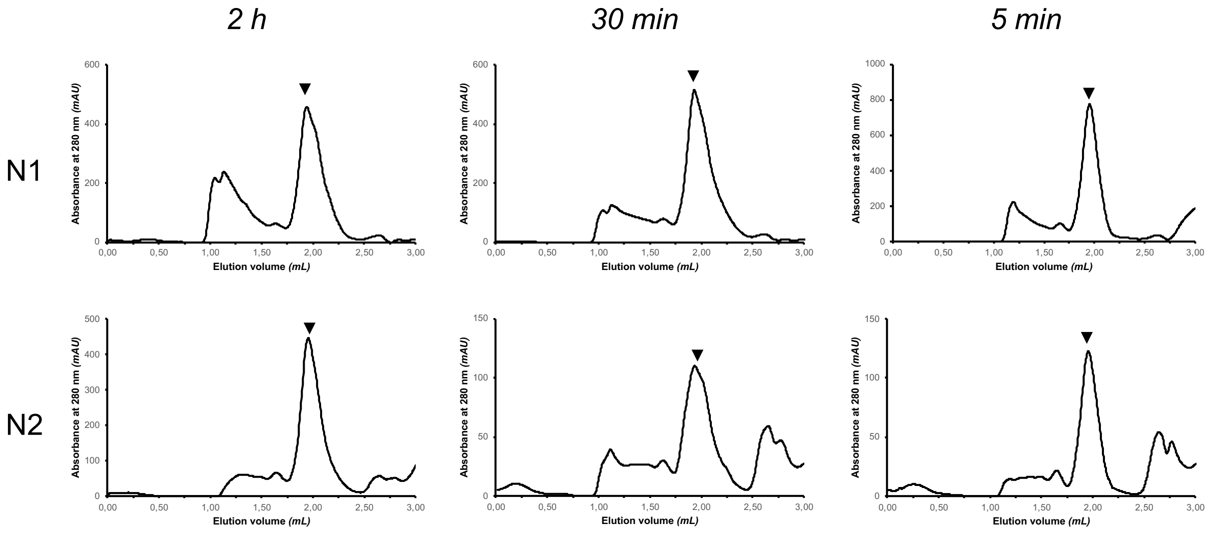

Supplement: Supplementary file 1 — Additional file 1. Corrected size-exclusion chromatograms. Prior to protein peak integration, spikes due to air bubbles were excluded. A: Chromatograms for analytical SEC of MBP-16E6mut. In order to control to oligomeric state of the purified protein, we performed systematic analytical SEC on the final protein sample. The calibration of the column allowed us to estimate the size of the particles at different elution volumes, thus the elution peak of monomeric protein is indicated by an arrow. Note the increase of the monomer fraction compared to the total amount of purified protein when shifting from the filter elution to the “decant and take up” elution method. B: Chromatograms for analytical SEC of MBP-8E6. On this protein challenging to purify, the increase of the monomer fraction (indicated by an arrow) between the two elution methods is dramatic. C: Chromatograms for analytical SEC of MBP-8E6: optimization of bacterial extract contact time. The elution peak of monomeric protein is indicated by an arrow. The decrease of the oligomeric fraction (eluted between the void volume V0 and the monomer fraction) is particularly visible for nickel resin N1. [file 12934_2018_1039_MOESM1_ESM.pdf]
